# *Vibrio alginolyticus* Associated Chronic Myringitis Acquired in Mediterranean Waters of Turkey

**DOI:** 10.1155/2015/187212

**Published:** 2015-10-29

**Authors:** Burak Ekrem Citil, Serhan Derin, Funda Sankur, Murat Sahan, Mahmut Ugur Citil

**Affiliations:** ^1^Department of Microbiology, Mugla Sitki Kocman University, 48000 Mugla, Turkey; ^2^Department of Otolaryngology, Mugla Sitki Kocman University, 48000 Mugla, Turkey; ^3^Department of Microbiology, Unye State Hospital, 52300 Ordu, Turkey

## Abstract

*Vibrio alginolyticus* was originally classified as biotype 2 of *Vibrio parahaemolyticus*. Most clinical isolates are recovered from superficial wounds or the external ear infections. *V. alginolyticus* is acknowledged to be nearly nonpathogenic in humans. The reason for presence of *V. alginolyticus*'s virulence is uncertain. We describe a chronic myringitis case in a 47-year-old female due to *V. alginolyticus*. According to her anamnesis, it was detected that she had sea bathing history in Mugla Coast in Turkey. Pure isolation of *V. alginolyticus* was obtained from external auditory canal's culture. Investigation and antimicrobial susceptibility of the isolate were performed by the automatized BD Phoenix system and Kirby-Bauer disk diffusion method, respectively. The bacteria were sensitive to all antibiotics. This case was presented to pay attention to *Vibrio alginolyticus* infections.

## 1. Introduction

*Vibrio alginolyticus* as a human pathogen is a rare factor in human infections [1]. These bacteria are a halophilic* Vibrio* species where they often can be isolated from the coastal flora of temperate seas and the rivers where they flow into the sea [2]. This agent, unlike other* Vibrio* species, rarely causes gastrointestinal tract infections and usually does superficial wound, ear (otitis media, otitis externa), and conjunctival infections [3]. Especially in the summer months, the incidence of infections caused by these bacteria is significantly increased [4]. Infections caused by* Vibrio alginolyticus* can usually be treated in a short time with the appropriate antibiotic selection, but in the literature, especially immune compromised patients, sphenoiditis, intracranial infections, necrotizing fasciitis, and even septic shock cases have been reported [1, 5–7].

In this paper, we describe a patient diagnosed with chronic myringitis in culture of whom* V. alginolyticus* was isolated from the external auditory canal. We intend to draw attention to* V. alginolyticus* infections that are rare causative pathogen in human infections especially ear cavity.

## 2. Case Report

Regarding 47-year-old female patient who has sea bathing history, left ear discharge complaint was present for nearly three months. In otoscopic examination, left modified radical mastoidectomy cavity (canal wall down tympanoplasty) was realized. Tympanic membrane was intact and 5 × 5 mm in diameter; granulation tissue was observed. This tissue was curetted and cauterized with silver nitrate following the swab culture sampling. Ciprofloxacin ear drops treatment was initiated empirically at a dose of 3 × 3.

Ear discharge sample in Stuart transport medium was inoculated into BAP and EMB agar media. The inoculated media were incubated at 37°C overnight. This resulted in a pure growth of a Gram-negative bacterium. The colonies had smooth, convex morphology and were creamy in consistency and gray with full edges on BAP agar. Isolated strain's oxidase test was positive. Based on Gram stained microscopy, colony morphology, oxidase test result, and case history, a pathogenic marine* Vibrio* was considered. The strain was initially identified as* V. alginolyticus by the* Phoenix 100 automated microbiology system (Becton Dickinson Diagnostics, USA). This bacterium grew on thiosulfate-citrate-bile salts-sucrose (TCBS) agar by forming yellow colonies after 24 hours of incubation. Isolate's fermentative characteristics were affirmed by inoculation in TSI (triple sugar iron agar) medium. Identification was also verified by conventional biochemical tests. The bacteria have been positive for indole, methyl red, Voges-Proskauer, and lysine decarboxylase tests and negative for growth on Simmons' citrate agar, H_2_S on TSI, gas production, urea hydrolysis, and arginine hydrolysis. Isolate's antibiotic susceptibility was tested by using Kirby-Bauer disk diffusion method. The zone diameters were interpreted according to CLSI guidelines [8]. The organism was susceptible to a variety of antibiotics including amoxicillin-clavulanate, cephalothin, cefuroxime, third-generation cephalosporins, aminoglycosides, ciprofloxacin, and trimethoprim-sulfamethoxazole. Considering antibiotic susceptibility results, the treatment was completed in 14 days with oral trimethoprim-sulfamethoxazole. At the end of treatment, she was seen as healthy. No growth was seen in control cultures.

## 3. Discussion

The incidence of infections caused by* Vibrio* species shows a positive correlation with sea water temperatures [9]. These species are salt-tolerant and can grow in hugely high salt concentrations (as high as 10%) and their virulence is related to their ability to produce hemolysis, hemagglutination, and protease [1, 10].* V. vulnificus* is the most virulent species of noncholera* Vibrios*. Actually,* V. alginolyticus* is considered comparatively nonpathogenic in humans in contrast to* V. vulnificus*. It has been rarely reported that* V. alginolyticus* causes infections [1]. Most clinical isolates are recovered from superficial wounds or external ear infections [11]. Conjunctivitis, acute gastroenteritis, bacteremia, and necrotising fasciitis caused by* V. alginolyticus* have also been reported [12]. Although cases are rarely reported from cold northern countries such as UK and Netherlands, the majority of cases reported in the literature belong to countries where they have warm sea coast like the Mediterranean countries [1, 2, 13–17].

Otitis media and otitis externa are common infections of upper respiratory tract. Ear infections that are caused by* V*.* alginolyticus* have similar clinical presentation with more often isolated pyogenic agents like* Pseudomonas aeruginosa, Staphylococcus aureus, Proteus mirabilis,* and* Escherichia coli*.

To the best of our knowledge, this is the fourth reported case of* V. alginolyticus* in Turkey. Two of these cases were acute external otitis cases [18, 19]. And the third one is* V. alginolyticus* bacteremia [20]. In both otitis cases, infection has emerged subsequently after they had holidays in the Mediterranean region like in our patient's history. Epithelial migration ability and being self-cleaning mechanism are weakened in mastoidectomy cavities. Also, opened temporal bone after mastoidectomy operation secretes tissue fluid, which is convenient for bacterial proliferation [21]. We think combination of these factors facilitated the infection of this halophilic* Vibrio* type in which presence of virulence remains unclear. On the other hand, anatomic reformation of the external auditory canal has reduced the effectiveness of the local antibiotic treatment and has allowed the chronicity of infection.

## 4. Conclusions

In the spring and summer months that the sea temperatures rise, this unusual causative pathogen should be noted for external ear and wound infections. So, especially in temperate regions, patients who admit to having otorrhea and skin infection symptoms should be questioned for sea bathing history. To identify the phenotype and to interpret the antibiotic susceptibility profiles of the microorganism, bacteriological culture samples must be taken before start of treatment. This property in the medical history should be shared with the microbiology laboratory in order to be guiding to define the pathogen. We believe that in particular these approaches are important to prevent the development of chronic ear infections.
